# Evolving data standards for cryo-EM structures

**DOI:** 10.1063/1.5138589

**Published:** 2020-01-24

**Authors:** Catherine L. Lawson, Helen M. Berman, Wah Chiu

**Affiliations:** 1 Institute for Quantitative Biomedicine and Research Collaboratory for Structural Bioinformatics, Rutgers, The State University of New Jersey, Piscataway, New Jersey 08854, USA; 2 Department of Chemistry and Chemical Biology, Rutgers, The State University of New Jersey, Piscataway, New Jersey 08854, USA; 3 Department of Biological Sciences and Bridge Institute, University of Southern California, Los Angeles, California 90089, USA; 4 Division of CryoEM and Biomaging, SLAC National Accelerator Laboratory, Stanford University, Stanford, California 94025, USA; 5 Department of Bioengineering, Stanford University, Stanford, California 94305, USA

## Abstract

Electron cryo-microscopy (cryo-EM) is increasingly being used to determine 3D structures of a broad spectrum of biological specimens from molecules to cells. Anticipating this progress in the early 2000s, an international collaboration of scientists with expertise in both cryo-EM and structure data archiving was established (EMDataResource, previously known as EMDataBank). The major goals of the collaboration have been twofold: to develop the necessary infrastructure for archiving cryo-EM-derived density maps and models, and to promote development of cryo-EM structure validation standards. We describe how cryo-EM data archiving and validation have been developed and jointly coordinated for the Electron Microscopy Data Bank and Protein Data Bank archives over the past two decades, as well as the impact of evolving technology on data standards. Just as for X-ray crystallography and nuclear magnetic resonance, engaging the scientific community via workshops and challenging activities has played a central role in developing recommendations and requirements for the cryo-EM structure data archives.

## INTRODUCTION

**FIG. 1.**
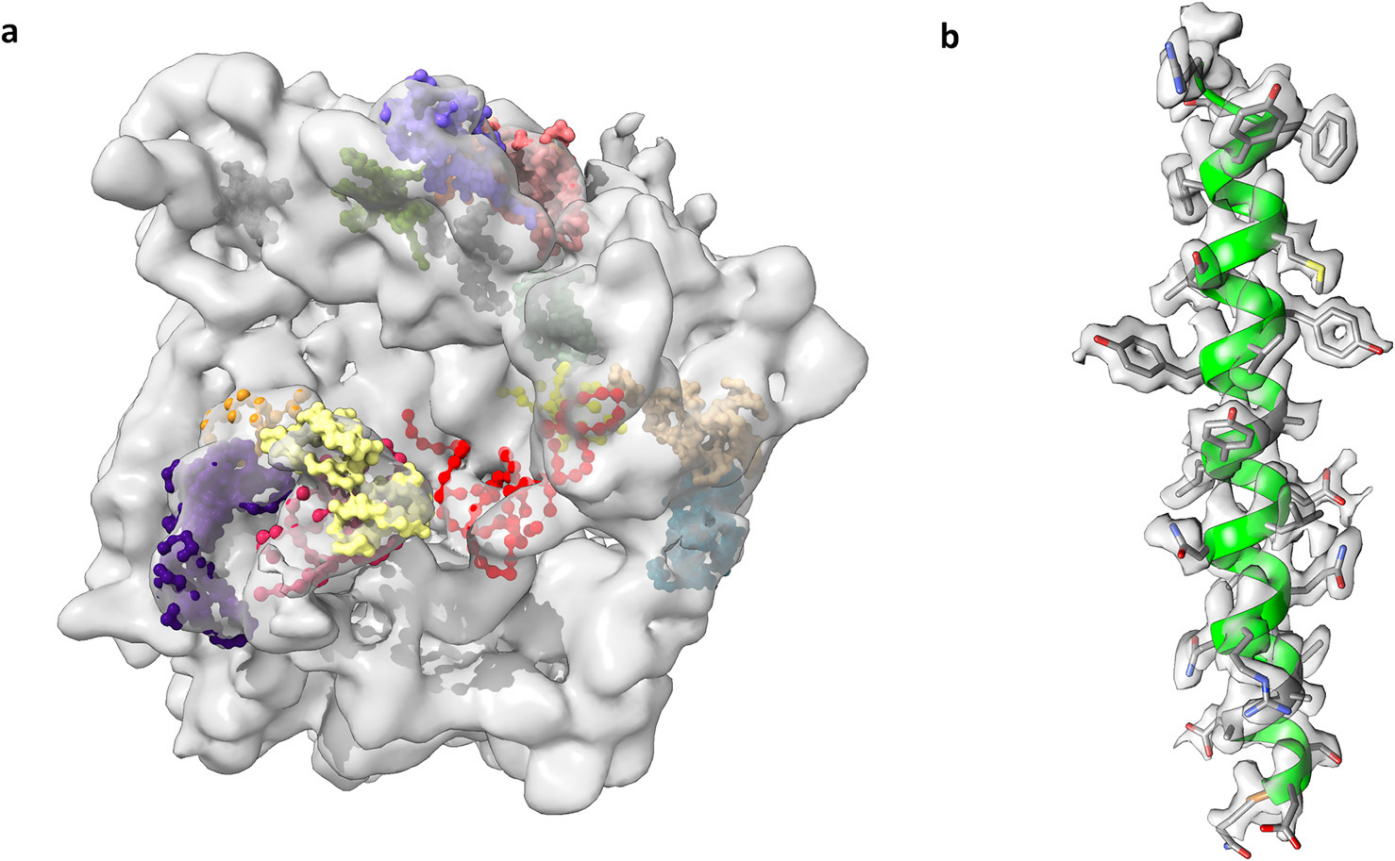
Cryo-EM: contrasting early (2000) vs recent (2019). (a) Cryo-EM structure of the *E. coli* 70S ribosome determined by the Frank group at 11.5 Å, one of the first maps deposited in the EMDB archive (EMD-1003). It is shown here superimposed with manually fitted components deposited to PDB (1eg0).^37^ (b) Helical segment of the 1.8 Å apoferritin map used as a target in the 2019 model challenge,^36^ with a fitted model.

Electron cryo-microscopy (cryo-EM) has very recently become a mainstream area of structural biology and medicine, enabling 3D visualization of a wide variety of biologically important complexes that were previously inaccessible to science. Early cryo-EM 3D density maps typically lacked atomic detail, yielding only the overall molecular shape, but could still sometimes be interpreted at a “pseudo-atomic” level via fitting of previously known coordinates or homology models [Fig. 1(a)].^1^ Recent major technological advances now make it increasingly possible to directly visualize atomic details [Fig. 1(b)].^2,3^ These achievements were recognized by the award of the 2017 Chemistry Nobel Prize to cryo-EM pioneers Dubochet, Frank, and Henderson.^4^

**FIG. 2.**
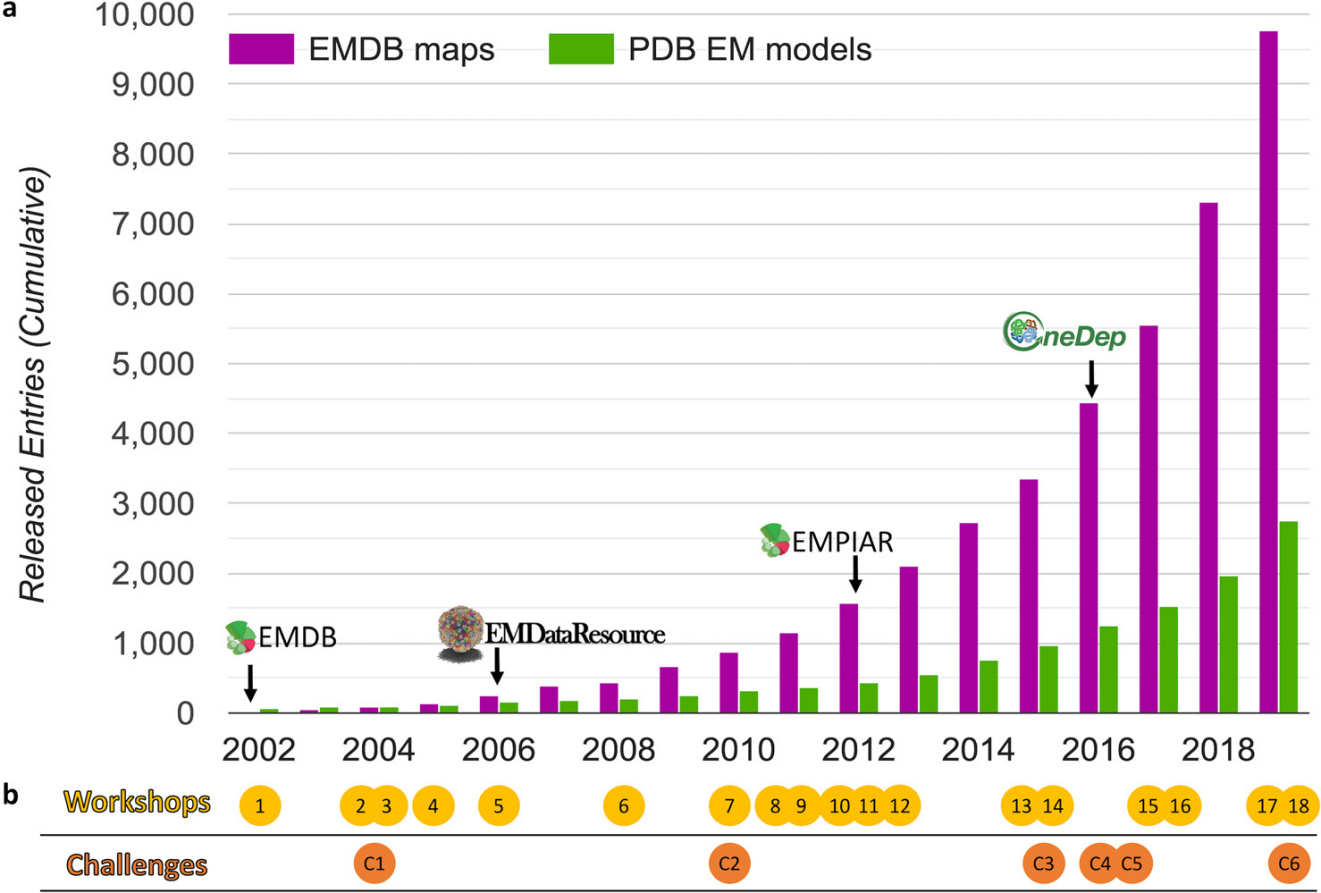
Growth of data archives and community activity timeline. (a) Released map entries in EMDB and released EM model coordinate entries in PDB are shown, cumulative by year. Milestones (indicated with arrows) are described in the main text. Plot source: emdataresource.org. (b) Workshops (yellow circles) and challenges (orange circles) related to data standards and validation development are plotted according to the year they were held. Numbers within the circles correspond to Tables II and III rows.

The development of cryo-EM is directly reflected by the growth of cryo-EM structure depositions contributed worldwide to public data archives [Fig. 2(a)]. The archiving systems and underlying data standards supporting deposition, annotation, release, and validation of cryo-EM structures and the associated metadata describing cryo-EM experiments have been developed over time to support this growth.^5^ We outline here the history of these systems and describe the process by which data standards have been developed, highlighting the role of engaging the scientific community to develop recommendations and requirements. The archiving systems and standards continue to evolve as technology drives the need for new descriptors and validation metrics.

## CRYO-EM STRUCTURE DATA ARCHIVING

**FIG. 3.**
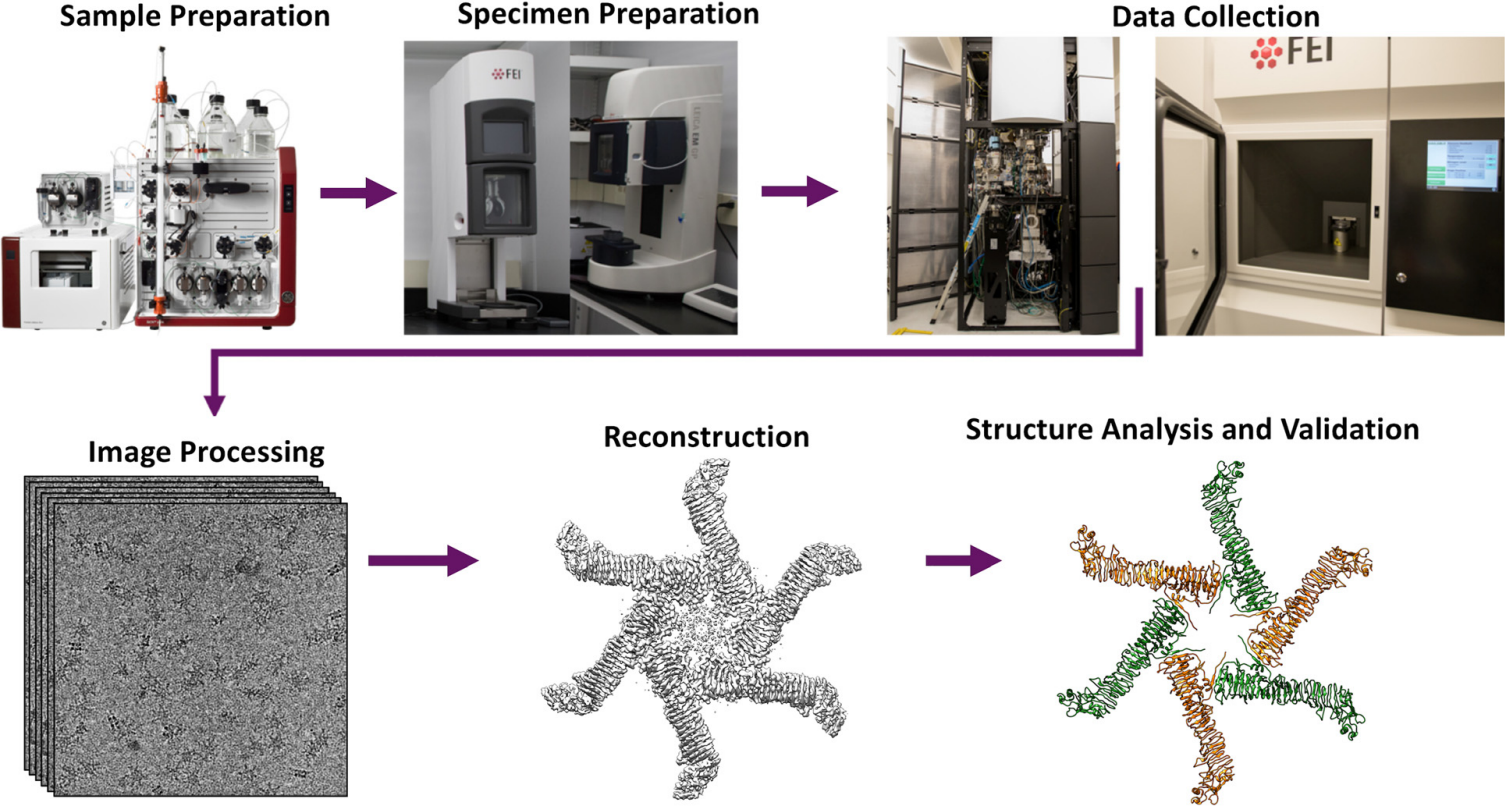
Cryo-EM experimental workflow. The major steps for determining a structure using cryo-EM single particle reconstruction are shown. The specimen shown is *Helicobacter pylori* vacuolating cytotoxin A oligomer.^38^

The Protein Data Bank (PDB), established in 1971 as a public archive for atomic coordinates of biological structures derived from X-ray crystallography,^6^ began accepting models derived from nuclear magnetic resonance spectroscopy (NMR) in 1988,^7^ and from electron microscopy (EM) and electron crystallography (EC) in 1990.^8^ In recognition of the fact that publicly available 3D density maps could accelerate discovery in structural biology and medicine, the Electron Microscopy Data Bank (EMDB) at the European Bioinformatics Institute (EBI) was launched in 2002 with support from the European Union.^9^ EMDB's launch was quickly followed by a pair of editorials in *Structure* and *Nature Structural Biology* encouraging electron microscopists to deposit their density maps.^10,11^ Similar to BioMagResBank (BMRB), which archives experimental data from NMR,^12^ the EMDB accepts maps determined using any cryo-EM method, including single particle reconstruction with any symmetry, helical filament reconstruction, subtomogram averaging, tomography, and electron crystallography, along with metadata describing the full experimental workflow (Fig. 3).

In 2006, scientists in the UK (EMDB) and USA [Research Collaboratory for Structural Bioinformatics (RCSB) and the National Center for Macromolecular Imaging (NCMI)] initiated a collaboration funded by National Institutes of Health (NIH) aimed to ensure that data archiving and validation standards for cryo-EM maps and models would be coordinated internationally.^13,14^ The project, formerly known as EMDataBank, was recently rebranded as EMDataResource (EMDR; emdataresource.org). The EMDR project website serves as a global resource for cryo-EM structure data archiving and retrieval, news, events, software tools, data standards, validation methods, and community challenges.

EMDep, designed and implemented at EBI, was the first system designed to collect and annotate maps and associated metadata for EMDB.^9^ In 2008, the EMDR team created a joint map+model deposition system for cryo-EM structures by connecting EMDep with AutoDep and ADIT (AutoDep Input Tool), the PDB data collection systems at the EBI and RCSB sites.^13^ The system that was implemented enabled a one stop shop for cryo-EM model and map depositions. Joint curation ensured that maps and models were deposited at the same physical scale and in the same coordinate frame. Journals that publish cryo-EM structures began to require authors to deposit maps to EMDB and models to PDB. This system supported the processing and release of nearly 4000 maps and 1000 models over a nine-year period (2008–2015).^14^

In 2012, the Electron Microscopy Public Image Archive (EMPIAR) was established at EBI.^15^ Supported by the UK Medical Research Council and UK Biotechnology & Biological Sciences Research Council, EMPIAR enables cryo-EM scientists to archive and share raw images and intermediate data files associated with their maps deposited to EMDB. Making recently collected image data broadly available has multiple benefits, including accelerating development of reconstruction software, and enriching resources for cryo-EM scientists in training. EMPIAR has its own deposition and curation system, but accesses metadata from the related EMDB entry. Individual entry storage sizes can be up to 15 TB. Approximately 4% of EMDB entries deposited since 2012 have associated EMPIAR entries.

**FIG. 4.**
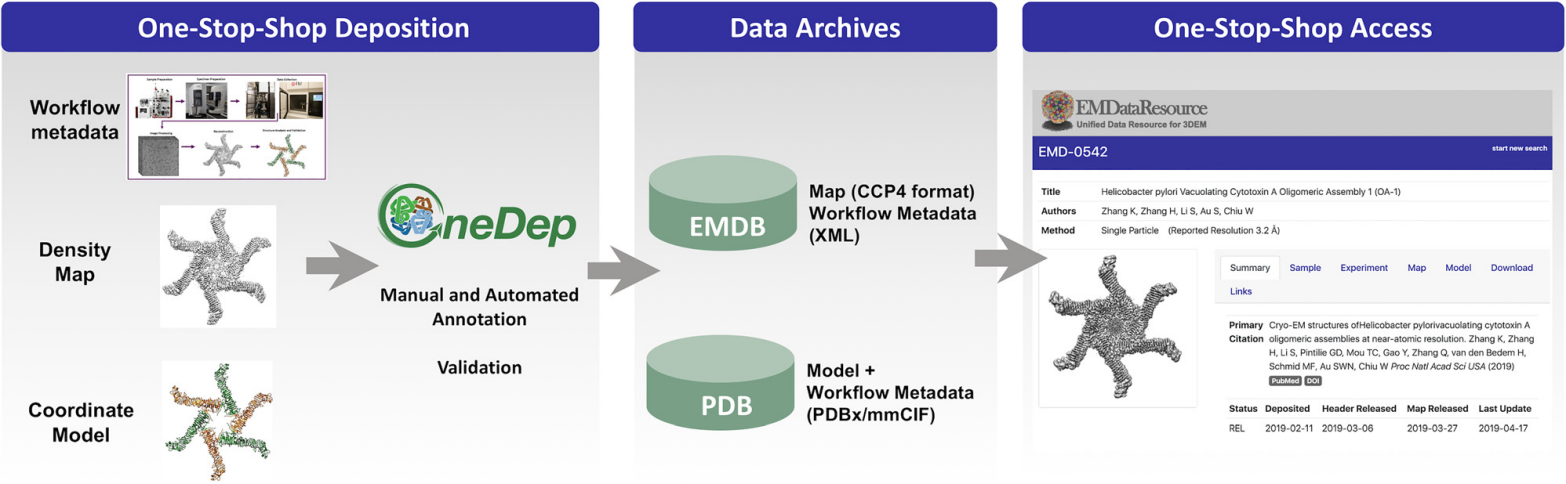
Current systems for deposition, archiving, and accessing cryo-EM structures. Worldwide, every cryo-EM structure (map, experimental metadata, and optionally coordinate model) is deposited and processed through the wwPDB OneDep system (deposit.wwpdb.org), following the same annotation and validation workflow also used for X-ray crystallography and NMR structures.^17,18^ Map-only depositions yield an EMDB entry, while joint map+model depositions yield both EMDB and PDB entries. Workflow metadata collected in OneDep are passed to both EMDB and PDB. EMDB holds all workflow metadata while PDB holds a subset of the metadata; see Table I. The PDB and EMDB archives are accessible by FTP and rsync at wwPDB mirror sites in the US, UK, and Japan. Released cryo-EM structure data from both archives can be accessed via EMDataResource, EMDB, and wwPDB partner websites.

The Worldwide PDB (wwPDB) is the global organization that manages the PDB archive.^16^ In 2016, deposition, annotation, and release of cryo-EM structure maps and models were migrated to the wwPDB OneDep system (Fig. 4), using requirements that were initiated and developed by EMDR.^17^ At that time, it became mandatory to deposit maps to EMDB for all cryo-EM models deposited to PDB. In addition, structure validation reports, which can be provided by depositors in an official PDF format to journal editors and reviewers as part of manuscript review, began to be produced for all cryo-EM structures.^18^

## CREATING A DATA DICTIONARY

The foundation of any data repository is its data representation scheme. Based in part on the International Union of Crystallography dictionary for small molecule crystallography Crystallographic Information File (CIF),^19^ the Macromolecular Crystallographic Information File (mmCIF) was developed in the 1990s to support rich data content for the macromolecular crystallographic experiment and its results, with precise data type definitions, logical groupings for related data items, explicit parent-child relationships, enumerations for controlled vocabulary, extensibility, and many other features embedded in a computer-readable format.^20^ This dictionary is now the Master Format for the PDB. Particularly relevant for cryo-EM, very large complexes are readily represented, since mmCIF has no limits on the number of atoms or polymer chains.

Following the lead of the crystallographic community, an mmCIF extension dictionary containing data terms for cryo-EM experiments was drafted jointly in the early 2000s based on requirements provided by the cryo-EM community. The dictionary was vetted and expanded by the scientific community via multiple workshops, and subsequently integrated by EMDR into the PDBx/mmCIF dictionary for use in the hybrid joint map+model deposition system.^13^ In 2015, based on feedback from additional workshops, the EMDR team further modified and expanded the dictionary in several ways. Hierarchical descriptions of complex specimens were enabled, and experimental descriptions for each of the cryo-EM methods were extended.^5^ The >500 term EM dictionary (Table I) is now the basis for cryo-EM depositions to both EMDB and PDB in the wwPDB OneDep system. The dictionary continues to be updated regularly to support the evolving needs of the scientific community.

**TABLE I.** PDBx/mmCIF EM Dictionary used by wwPDB OneDep. As described in Fig. 4, all workflow metadata categories are collected by the OneDep system. Most categories are archived in both PDB and EMDB; asterisked categories are archived only in EMDB.

*Top level*	*Sample/specimen preparation*	Image *processing & reconstruction*	Experimental *data*
em_experiment	em_buffer	em_3d_reconstruction	
em_software	em_buffer_component	em_image_processing	em_map^*^
	em_crystal_formation	em_particle_selection	em_structure_factors^*^
*Sample description*	em_embedding	em_volume_selection	em_layer_lines^*^
em_entity_assembly	em_sample_support	em_ctf_correction	
em_entity_assembly_molwt	em_specimen		
em_entity_assembly_naturalsource	em_staining	em_2d_crystal_entity	
em_entity_assembly_recombinant	em_vitrification	em_3d_crystal_entity	
em_virus_entity		em_helical_entity	
em_virus_natural_host		em_single_particle_entity	
em_virus_shell	em_fiducial_markers^*^		
	em_focused_ion_beam^*^	em_euler_angle_assignment^*^	
*Data collection*	em_grid_pretreatment^*^	em_final_classification^*^	
em_diffraction	em_high_pressure_freezing^*^	em_start_model^*^	
em_diffraction_shell	em_shadowing^*^		
em_diffraction_stats	em_support_film^*^	Structure *analysis*	
em_image_recording	em_tomography^*^	em_3d_fitting	
em_image_scans	em_tomography_specimen^*^	em_3d_fitting_list	
em_imaging	em_ultramicrotomy^*^	em_fsc_curve^*^	
em_imaging_optics			

## GATHERING COMMUNITY REQUIREMENTS

Developing a trusted scientific data repository requires careful attention to the interplay among science, technology, and community interest.^21^ Workshops and Challenges are two types of community outreach activities that are effective in bringing these three elements together; both have been employed multiple times to move EM data and validation standard development forward [Fig. 2(b)]. Workshops (typically 2–3 days) enable groups of experts to review current practices and develop recommendations, while Challenges (taking place over several months to a year) provide forums for experts to exercise and demonstrate current workflows and test novel procedures. Challenges can incorporate one or more workshops for planning or results review. Tables II and III list and summarize goals and outcomes of 18 international workshops and six challenges held over the past two decades. Below we provide additional descriptions of selected activities, as well as a montage of workshop photos (Fig. 5).

**TABLE II.** Cryo-EM community data archiving and validation workshops.

#	Year	Title/location	Organizers	Description	Key outcomes
1	2002	IIMS Workshop, UK	Kim Henrick, José-María Carazo, Stephen Fuller	Promote software development in the field of 3DEM molecular structure determination	Guidelines and release policies for the new EMDB archive.^10,11,39^ Deposition system to collect EMDB data and maps^9^
2	2004	3DEM Developers workshop, UK	Kim Henrick	Developer review of tools and software practices used in the field of cryoEM	Priorities developed for EMDB including electron tomography, PDB-EMDB cross-referencing, lossless map conversion, review of community map standards and conventions
3	2004	Cryo-EM Structure Deposition Workshop, NJ, USA	Helen Berman, Wah Chiu, Michael Rossmann	Develop community consensus on data items needed for deposition of maps and atomic models derived from cryoEM	Need for deposition one-stop-shop articulated. Recommendations incorporated in extended EM data dictionary^13^
4	2005	3DEM Developers Workshop, UK	Kim Henrick	Introduced EM data dictionary to software developers to facilitate its integration into 3DEM packages and electronic notebooks	Agreement to adopt a common set of conventions for maps^22^
5	2006	CryoEM Standards Task Force, TX, USA	Wah Chiu, David Belnap, José-María Carazo	Gather cryoEM map conventions and formats with associated metadata used by different developers	Key conventions (e.g., Euler angles) were evaluated for interoperability and conversion tools were created^40,41^
6	2008	Electron Crystallography Data Model Workshop, CA, USA	Wah Chiu, Cathy Lawson	Gather expert advice on metadata items in the EM dictionary pertaining to electron crystallography	Recommendations incorporated into EM data dictionary^13^
7	2010	EM Validation Task Force, NJ, USA	Helen Berman, Wah Chiu, Gerard Kleywegt, Cathy Lawson	Expert review of potential validation standards for maps and models produced by 3DEM reconstruction	Recommendations summarized in white paper,^25^ and implemented in OneDep validation reports^14,18^
8	2011	Model Challenge Workshop, HI, USA	Steve Ludtke, Cathy Lawson, Gerard Kleywegt, Helen Berman, Wah Chiu	Computational groups described and compared tools they used to model a selected set of published cryoEM structures	Results published in *Biopolymers* journal special issue^42^
9	2011	Data Management Challenges in 3DEM, UK	Ardan Patwardhan, Gerard Kleywegt, Jason Swedlow	Gather expert advice on key topics in data archiving and validation for 3DEM data, including data model, validation, raw-data archiving	Recommendations summarized in white paper.^23^ Web-based visualization tools developed.^28^ EMPIAR raw data archive created.^15^ Extended data model implemented^17^
10	2012	3DEM Modeling Workshop, TX, USA	Wah Chiu	Current challenges in creating and validating coordinate models built into cryo-EM maps	Recommendations gathered for modeling and validation standards and future model challenges
11	2012	3D Cellular Context for the Macromolecular World, UK	Ardan Patwardhan, Gerard Kleywegt, Jason Swedlow	Discussions on data archiving and validation for emerging 3D cellular imaging techniques	Recommendations summarized in white paper^24^
12	2012	Instruct Image Processing Center Developer Workshop, Spain	José-María Carazo	Effort to standardize information exchange in single particle reconstruction and to establish algorithm benchmarking	CTF benchmarking challenge^43^ and EMX convention for CTF and single-particle parameters developed^44^
13	2015	CryoEM Model Validation Workshop, MA, USA	Wah Chiu, Cathy Lawson, Paul Adams	Modeling experts met to present and discuss challenges in modeling into cryoEM maps	Gathered recommendations^5^ directly used in development of 2016 Model Challenge
14	2015	Building Bridges between Cellular and Molecular Structural Biology, UK	Ardan Patwardhan, Gerard Kleywegt	Expert discussions on how to integrate structural data from a diverse range of public archives covering cellular and molecular structural biology	Recommendations to develop tools/file formats for map segmentation, and tools to support biological structure annotation described in white paper^45^
15	2017	Model Challenge Assessment, LA, USA	Wah Chiu, Cathy Lawson, Paul Adams	First pass analyses of models and data submitted to the 2016 Model Challenge	Recommended metrics implemented on model challenge comparison website^46^
16	2017	CryoEM Structure Joint Challenges Workshop, CA, USA	Cathy Lawson, Wah Chiu	Joint review of the 2016 Map and Model Challenge activities	Results described in *Journal of Structural Biology* special issue;^30^ also featured in *Nature Methods* editorial^47^
17	2019	Frontiers in cryo-EM Validation, UK	Gerard Kleywegt, Garib Murshudov, Elena Orlova, Ardan Patwhardhan, Alan Roseman, Peter Rosenthal, Maya Topf, Martyn Winn	Discuss current and future community needs/challenges for validation tools to support maps and models from single-particle analysis	Meeting featured in 2018 Nature editorial.^48^ Recommendations white paper is in preparation
18	2019	Model Metrics Workshop, CA, USA	Cathy Lawson, Wah Chiu	Review modeling processes and assessment results of the 2019 Model Metrics Challenge and plan for future events	Recommendations editorial and full event manuscript are in preparation

**TABLE III.** Cryo-EM community challenge activities.

#	Year	Event	Organizing group(s)	Description	Key outcomes
C1	2004	Particle Picking Challenge	National Resource for Automated Molecular Microscopy (La Jolla)	Compare diverse particle selection algorithms	Algorithms from 12 developer groups were compared and contrasted^49^
C2	2010	Model Challenge	EMDataResource, NCMI	Computational groups were asked to apply their tools to a selected set of published cryoEM structures.	Results published in *Biopolymers* journal special issue^42^
C3	2015	CTF Challenge	Instruct Image Processing Center (Madrid), NCMI	Evaluate community/algorithm abilities to estimate key parameters of EM Contrast Transfer Function for a wide set of experimental conditions	CTF benchmarking challenge summary and results published^43^
C4	2016	Map Challenge	EMDataResource	Establish benchmark datasets, develop best practices, evolve criteria for validation of 3DEM reconstructions	Results described in *Journal of Structural Biology* special issue;^30^ also featured in *Nature Methods* Editorial^47^
C5	2016	Model Challenge	EMDataResource	Establish benchmark datasets, develop best practices, evolve criteria for validation of 3DEM map-derived models	Results described in *Journal of Structural Biology* special issue;^30^ also featured in *Nature Methods* Editorial^47^
C6	2019	Model Metrics Challenge	EMDataResource	Identify metrics most suitable for evaluating/comparing fit of atomic coordinate models into cryo-EM maps in 1.8–3.0 Å resolution range	Recommendations editorial and full event manuscript are in preparation

**FIG. 5.**
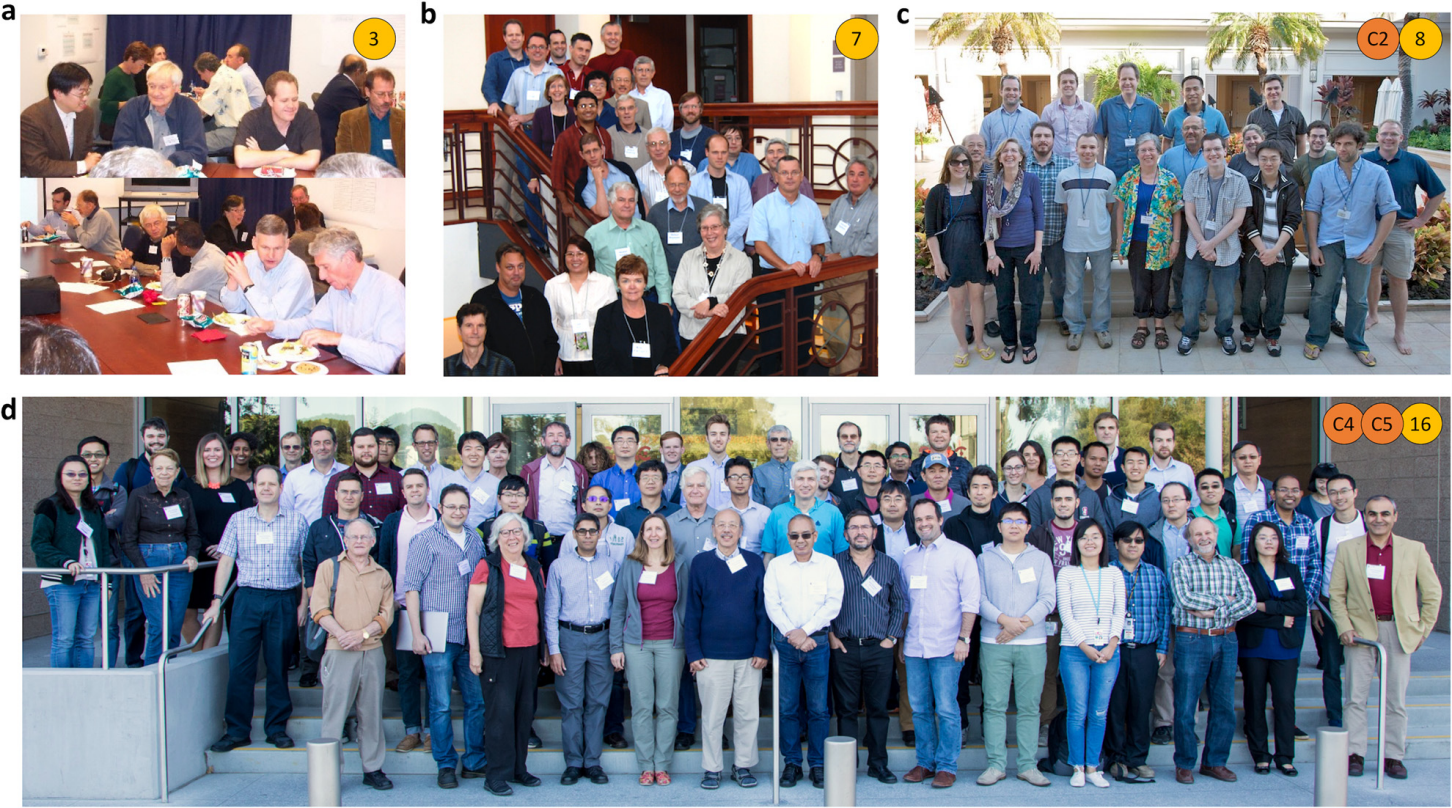
Workshop Participant Photos. (a) 2004 Cryo-EM structure deposition workshop; (b) 2010 EM Validation Task Force (EM VTF) Workshop; (c) 2011 model challenge workshop; (d) 2017 joint challenges workshop. Image in (d) Reprinted with permission from C. L. Lawson and W. Chiu, J. Struct. Biol. **204**(3), 523–526 (2018). Copyright 2018 Elsevier.

### EM extension dictionary development

**FIG. 6.**
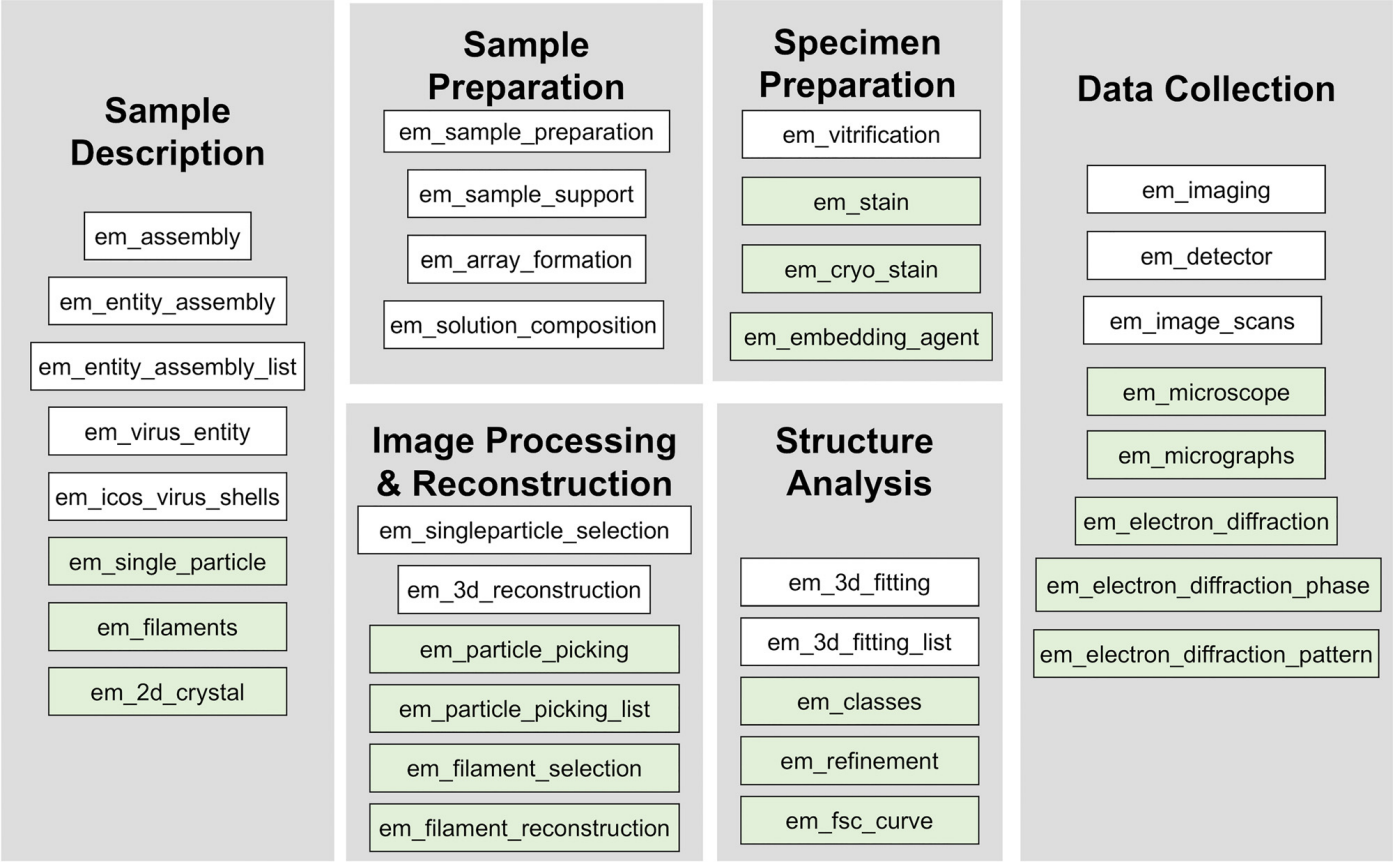
Overall structure of the EM extension data dictionary circa 2005. New categories of data items recommended by participants of the *2004 Cryo-EM Structure Deposition Workshop* are shown in green.

The main goal of the *2004 Cryo-EM Structure Deposition Workshop* [Fig. 5(a)], attended by ∼30 scientists including cryo-EM, image processing, crystallography, database, funding agency, and journal representatives, was to develop a global community consensus on data items for deposition of density maps and atomic models derived from cryo-EM studies. Terms were reviewed category-by-category in two focus groups, and recommendations for revisions and extensions were obtained (Fig. 6). Furthermore, participants unanimously requested a “one-stop shop” for deposition and retrieval of the cryo-EM map and model data. Following the workshop, the dictionary was further revised with follow-up input from attendees. The resulting dictionary was presented at the 2005 3DEM Gordon Research Conference, and EMDR's project website became the requested one-stop-shop portal.

The EM extension dictionary was next reviewed by software developers at the *2005 3DEM Developers Workshop* to facilitate its integration with major 3DEM packages and electronic notebook systems. There were two important outcomes: (a) the draft dictionary was unanimously accepted by the participants and (b) a set of proposed conventions for describing EM micrographs and density maps was developed.^22^ The conventions enable a standardized approach to image interpretation and presentation, with recommended units for common parameters, rotation and symmetry notations, and common sense principles such as “objects should have overall positive density” (early image correction procedures sometimes generated objects darker than their background depending on image processing and display software). The conventions were subsequently incorporated into the EM extension dictionary to facilitate representation of map-related data items in PDB and EMDB.

Data standards for cryo-EM structures were further addressed at the *2011 Data Management Challenges in 3D Electron Microscopy Workshop*^23^ and the *2015 Building Bridges between Cellular and Molecular Structural Biology Workshop*.^24^ Needs for hierarchical sample description as well as extensions to cryo-EM experimental sub-method descriptions were recognized. A future archival segmentation file format, for which requirements were gathered at the 2015 meeting, will make use of the hierarchy, enabling map regions to be connected with biological annotations.^5,24^

### Developing validation standards

At the *2010 EM Validation Task Force (EM VTF) Workshop* [Fig. 5(b)], an international group of experts explored how to assess cryo-EM maps, models, and other data deposited into EMDB and PDB. For maps, participants recognized a critical need to develop standards for assessing map resolution and accuracy. They recommended establishing two fully independent image datasets at the outset for evaluating resolution by Fourier Shell Correlation (FSC); at the time, this was not typically done, but it is now the standard procedure. However, they also advised that maps still be carefully inspected to ensure that the resolution estimate by FSC is in accordance with the map's visible features.

The EM VTF's 2012 white paper notably called for the scientific community to develop new criteria for the evaluation of maps and for the evaluation of fit of the model to the experimental map density.^25^ In contrast, in 2011 the VTF for X-ray crystallography published a comprehensive and detailed set of recommendations to validate structures and experimental data determined using X-ray crystallography.^26^ The difference reflects the fact that cryo-EM is still a rapidly evolving field.

Validation standards and raw image data archiving were additional topics of discussion at the *2011 Data Management Challenges in 3D Electron Microscopy Workshop*.^23^ Several services were developed and implemented at EBI in response to workshop recommendations. The EMPIAR raw data archive was created,^15^ and stand-alone FSC and tilt-pair servers were developed for depositors to validate their cryo-EM maps.^5,27^ In addition, Visual Analysis web pages were designed to display an informative series of images and plots for every EMDB entry, and to help users assess data quality of released cryo-EM maps and models.^28,29^

Two EMDR-sponsored challenges subsequently aimed to address the 2010 EM VTF's call for improved metrics to evaluate both maps and fit of models to experimental data (*2016 Map and Model Challenges)*. Following the *2017 Joint Challenges Workshop* at Stanford, which had over 90 participants [Fig. 5(d)]; key results and recommendations were collated into a virtual special issue of the *Journal of Structural Biology* published in December 2018.^30^

**FIG. 7.**
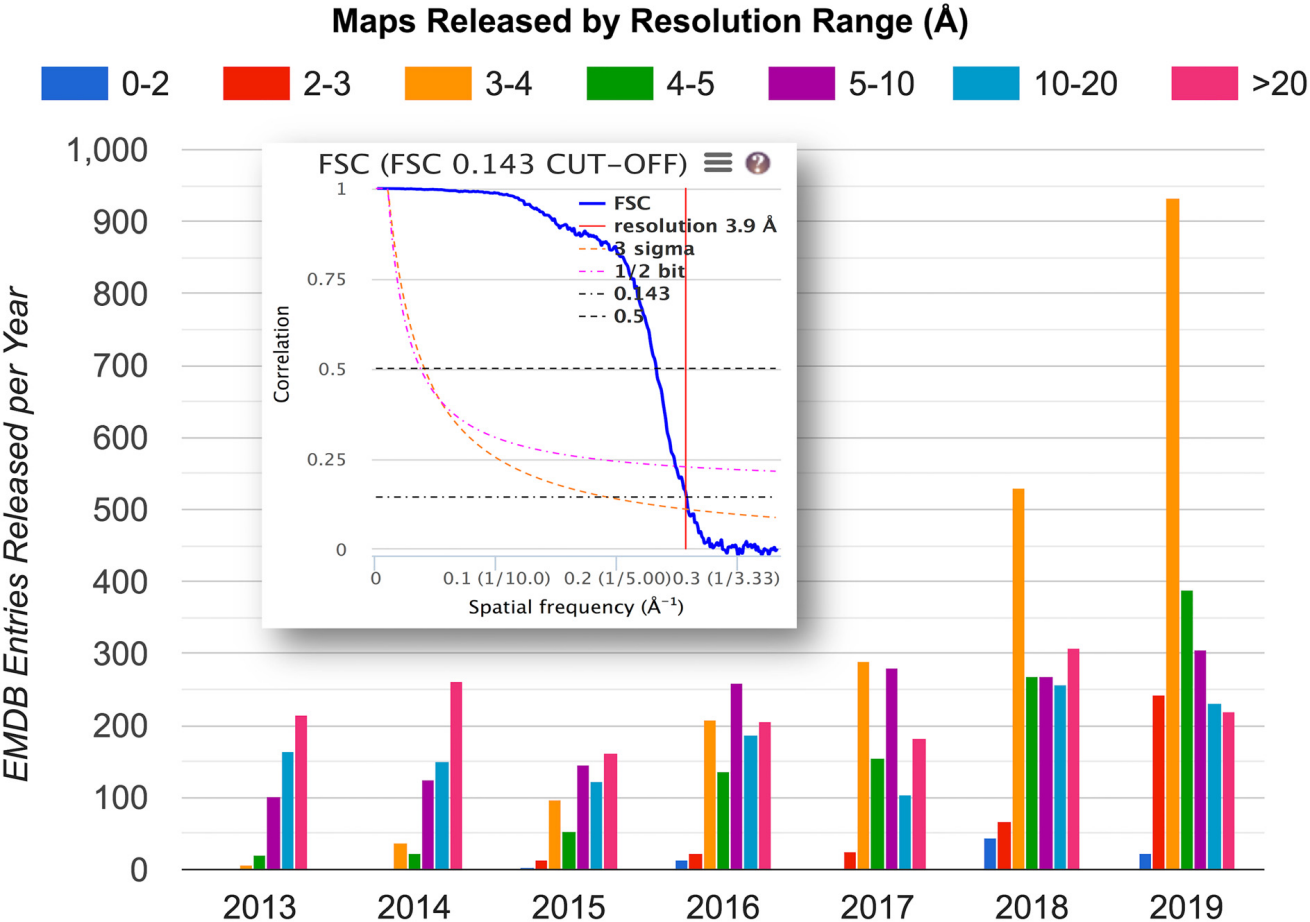
Changing cryo-EM resolution landscape. Annual distribution of depositor-reported resolution for map entries released into EMDB. The sharp increase at 2–4 Å resolution is a direct consequence of the recent advances in image detection and processing.^3^ Inset: example Fourier Shell Correlation (FSC) plot, which is the current standard for estimating map resolution.^25^ The correlation between two independent half-map reconstructions (blue curve) falls with decreasing spatial frequency; the resolution estimate (in this case 3.9 Å) is read at FSC = 0.143 (dash-dotted horizontal line). Plot source: emdataresource.org. Inset FSC plot source: EMDB visual analysis.^28^

The Map Challenge provided a unique forum for critically evaluating the standard method for estimating map resolution by FSC (Fig. 7, inset). A key observation was that as currently practiced, the procedure is not sufficiently standardized: a number of different variables (e.g., map box size, voxel size, filtering and masking practice, and threshold value for interpretation) can substantially impact the outcome.^31^ As a result, different expert practitioners can arrive at different resolution estimates for the same level of map details. For example, two of the apoferritin maps submitted to the challenge had practitioner-estimated resolutions of 3.1 Å and 3.5 Å, respectively, though they were indistinguishable by eye. A direct conclusion is that any “reported-resolution”-based search or ranking for maps or associated models will have limited reliability. In follow-up discussions at the *2019 Frontiers in Cryo-EM Validation Workshop*, one suggestion made was to have the archives independently estimate resolution by FSC from deposited unmasked, minimally filtered half-maps. This procedure would likely make comparisons between maps less susceptible (though not completely impervious) to variations in practitioner practice.

The *2017 Joint Challenges Workshop* also sparked lively discussions about the potential for model-based metrics to estimate not only model quality, but also to provide one or more independent measures of map resolvability. Several procedures of this type have been proposed and tested. EMRinger evaluates whether density peaks in the map fall within the possible rotameric configuration for the carbon-β atom in a side chain.^32^ Other procedures have been developed to measure map quality. For example, Z-scores capture how much larger the cross-correlations score (CCS) is for atoms in such features at their placed location compared to the CCS at displaced positions.^33–35^ Another recently devised experimental metric, Q-score, measures resolvability of the individual atom(s) in reference to the model.^36^

### Changing validation goals

Looking at the distribution of reported resolution of maps released into EMDB annually over the past few years (Fig. 7), one can readily see a striking sharp recent increase in maps in the 2–4 Å range. This development is a direct result of recent technological improvements, and it changes the “goal-posts” for developing validation methods, adding urgency to the need for metrics to validate structures at near-atomic to atomic resolution.

The *2019 Model Metrics Challenge* and associated *2019 Model Metrics Workshop* were designed with the goal of evaluating metrics for map-model fit of moderately high-resolution maps (3.1–1.8 Å). A full write-up will be published elsewhere, and we note two findings here. First, the new metrics that by some means combine both model and map quality (e.g., EMRinger and Q-score) appear to be quite useful for ranking sets of structures. Second, refined Atomic Displacement Parameters (ADPs), which were included in about half of the models submitted by challenge participants, could modestly improve fit of the model to the map, particularly for the highest resolution (1.8 Å) target map. The meaning of refined ADPs/B-factors in the context of a cryo-EM density map is less clear. Best practices (e.g., to avoid overfitting) will need to be investigated.

## WHERE WE ARE, WHAT'S NEXT

The initial EM validation report format released in 2016 focused on assessment of model geometry for PDB entries.^18^ As will be reported in more detail in a future publication, additional sections covering map analysis and visualization and map-model fit analysis and visualization will become available to EMDB and PDB depositors by early 2020. The Visual Analysis web pages hosted at EBI since 2012^28^ have served as a test-bed for the development of the new features, which will include (a) several types of orthogonal images of the deposited map and map superimposed with model; (b) FSC curves to support depositor-reported map resolution; and (c) map-model fit statistics via “atom inclusion,” the percentage of modeled atoms falling inside a map at its recommended contour level. The new features will enable scientists (depositors, annotators, journal editors, and manuscript reviewers) to make initial assessments of map features, map quality, and map-model fit, bypassing the need to first download/view files in a graphics program.

A planned meeting in January 2020 at EBI organized by wwPDB will bring together cryo-EM and data archiving experts to discuss the current state of data archiving for cryo-EM structures derived from the single-particle reconstruction method, and to solicit recommendations on what data should be included and/or made mandatory in depositions and associated validation reports. The following points might be considered as part of the deliberations:

• Can estimation of map resolution be better standardized across the community? This would enable fairer comparisons among maps determined in different laboratories and using different software packages.
• Additional metrics (beyond atom inclusion) are available that describe map-model fit, including several novel procedures that effectively yield a joint assessment of map and model quality in a broad resolution range. How should map-model fit be reported as part of a structure determination and in a joint map+model deposition?
• What best practice recommendations can be made for refinement of ADPs in cryo-EM models at different resolutions?
• How should we evaluate multiple structures determined from a single specimen that may have variable quality and resolution?

## References

[1] W. Chiu , M. L. Baker , W. Jiang , M. Dougherty , and M. F. Schmid , “ Electron cryomicroscopy of biological machines at subnanometer resolution,” Structure 13(3), 363–372 (2005). 10.1016/j.str.2004.12.016 15766537

[2] K. R. Vinothkumar and R. Henderson , “ Single particle electron cryomicroscopy: Trends, issues and future perspective,” Q. Rev. Biophys. 49, e13 (2016). 10.1017/S0033583516000068 27658821

[3] W. Kuhlbrandt , “ Biochemistry. The resolution revolution,” Science 343(6178), 1443–1444 (2014). 10.1126/science.1251652 24675944

[4] Y. Cheng , R. M. Glaeser , and E. Nogales , “ How cryo-EM became so hot,” Cell 171(6), 1229–1231 (2017). 10.1016/j.cell.2017.11.016 29195065 PMC6186021

[5] A. Patwardhan and C. L. Lawson , “ Databases and archiving for CryoEM,” Methods Enzymol. 579, 393–412 (2016). 10.1016/bs.mie.2016.04.015 27572735 PMC5351295

[6] “Crystallography: Protein data bank,” Nat. New Biol. **233**(42), 223–223 (1971).

[7] P. C. Driscoll , A. M. Gronenborn , L. Beress , and G. M. Clore , “ Determination of the three-dimensional solution structure of the antihypertensive and antiviral protein BDS-I from the sea anemone Anemonia sulcata: A study using nuclear magnetic resonance and hybrid distance geometry-dynamical simulated annealing,” Biochemistry 28(5), 2188–2198 (1989). 10.1021/bi00431a033 2566326

[8] R. Henderson , J. M. Baldwin , T. A. Ceska , F. Zemlin , E. Beckmann , and K. H. Downing , “ Model for the structure of bacteriorhodopsin based on high-resolution electron cryo-microscopy,” J. Mol. Biol. 213(4), 899–929 (1990). 10.1016/S0022-2836(05)80271-2 2359127

[9] K. Henrick , R. Newman , M. Tagari , and M. Chagoyen , “ EMDep: A web-based system for the deposition and validation of high-resolution electron microscopy macromolecular structural information,” J. Struct. Biol. 144(1–2), 228–237 (2003). 10.1016/j.jsb.2003.09.009 14643225

[10] S. D. Fuller , “ Depositing electron microscopy maps,” Structure 11(1), 11–12 (2003). 10.1016/S0969-2126(02)00942-5 12517335

[11] “Editorial, A database for ‘em’,” Nat. Struct. Biol. **10**(5), 313 (2003). 12714996 10.1038/nsb0503-313

[12] E. L. Ulrich , H. Akutsu , J. F. Doreleijers , Y. Harano , Y. E. Ioannidis , J. Lin , M. Livny , S. Mading , D. Maziuk , Z. Miller , E. Nakatani , C. F. Schulte , D. E. Tolmie , R. K. Wenger , H. Yao , and J. L. Markley , “ BioMagResBank,” Nucl. Acids Res. 36, D402–408 (2007). 10.1093/nar/gkm957 17984079 PMC2238925

[13] C. L. Lawson , M. L. Baker , C. Best , C. Bi , M. Dougherty , P. Feng , G. van Ginkel , B. Devkota , I. Lagerstedt , S. J. Ludtke , R. H. Newman , T. J. Oldfield , I. Rees , G. Sahni , R. Sala , S. Velankar , J. Warren , J. D. Westbrook , K. Henrick , G. J. Kleywegt , H. M. Berman , and W. Chiu , “ EMDataBank.org: Unified data resource for CryoEM,” Nucl. Acids Res. 39, D456–464 (2011). 10.1093/nar/gkq880 20935055 PMC3013769

[14] C. L. Lawson , A. Patwardhan , M. L. Baker , C. Hryc , E. S. Garcia , B. P. Hudson , I. Lagerstedt , S. J. Ludtke , G. Pintilie , R. Sala , J. D. Westbrook , H. M. Berman , G. J. Kleywegt , and W. Chiu , “ EMDataBank unified data resource for 3DEM,” Nucl. Acids Res. 44(D1), D396–403 (2016). 10.1093/nar/gkv1126 26578576 PMC4702818

[15] A. Iudin , P. K. Korir , J. Salavert-Torres , G. J. Kleywegt , and A. Patwardhan , “ EMPIAR: A public archive for raw electron microscopy image data,” Nat. Methods 13(5), 387–388 (2016). 10.1038/nmeth.3806 27067018

[16] wwPDB Consortium, “ Protein Data Bank: The single global archive for 3D macromolecular structure data,” Nucl. Acids Res. 47(D1), D520–D528 (2019). 10.1093/nar/gky949 30357364 PMC6324056

[17] J. Y. Young , J. D. Westbrook , Z. Feng , R. Sala , E. Peisach , T. J. Oldfield , S. Sen , A. Gutmanas , D. R. Armstrong , J. M. Berrisford , L. Chen , M. Chen , L. Di Costanzo , D. Dimitropoulos , G. Gao , S. Ghosh , S. Gore , V. Guranovic , P. M. S. Hendrickx , B. P. Hudson , R. Igarashi , Y. Ikegawa , N. Kobayashi , C. L. Lawson , Y. Liang , S. Mading , L. Mak , M. S. Mir , A. Mukhopadhyay , A. Patwardhan , I. Persikova , L. Rinaldi , E. Sanz-Garcia , M. R. Sekharan , C. Shao , G. J. Swaminathan , L. Tan , E. L. Ulrich , G. van Ginkel , R. Yamashita , H. Yang , M. A. Zhuravleva , M. Quesada , G. J. Kleywegt , H. M. Berman , J. L. Markley , H. Nakamura , S. Velankar , and S. K. Burley , “ OneDep: Unified wwPDB system for deposition, biocuration, and validation of macromolecular structures in the PDB archive,” Structure 25(3), 536–545 (2017). 10.1016/j.str.2017.01.004 28190782 PMC5360273

[18] S. Gore , E. Sanz Garcia , P. M. S. Hendrickx , A. Gutmanas , J. D. Westbrook , H. Yang , Z. Feng , K. Baskaran , J. M. Berrisford , B. P. Hudson , Y. Ikegawa , N. Kobayashi , C. L. Lawson , S. Mading , L. Mak , A. Mukhopadhyay , T. J. Oldfield , A. Patwardhan , E. Peisach , G. Sahni , M. R. Sekharan , S. Sen , C. Shao , O. S. Smart , E. L. Ulrich , R. Yamashita , M. Quesada , J. Y. Young , H. Nakamura , J. L. Markley , H. M. Berman , S. K. Burley , S. Velankar , and G. J. Kleywegt , “ Validation of structures in the Protein Data Bank,” Structure 25(12), 1916–1927 (2017). 10.1016/j.str.2017.10.009 29174494 PMC5718880

[19] S. R. Hall , F. H. Allen , and I. D. Brown , “ The crystallographic information file (Cif)—A new standard archive file for crystallography,” Acta Crystallogr., Sect. A 47, 655–685 (1991). 10.1107/S010876739101067X

[20] P. M. D. Fitzgerald , J. D. Westbrook , P. E. Bourne , B. McMahon , K. D. Watenpaugh , and H. M. Berman , in *International Tables for Crystallography G. Definition and Exchange of Crystallographic Data*, edited by Hall S. R. and McMahon B. ( Springer, Dordrecht, The Netherlands, 2005), pp. 295–443.

[21] H. M. Berman , C. L. Lawson , B. Vallat , and M. J. Gabanyi , “ Anticipating innovations in structural biology,” Q. Rev. Biophys. 51, e8 (2018). 10.1017/S0033583518000057 30912485 PMC6438187

[22] J. B. Heymann , M. Chagoyen , and D. M. Belnap , “ Common conventions for interchange and archiving of three-dimensional electron microscopy information in structural biology,” J. Struct. Biol. 151(2), 196–207 (2005). 10.1016/j.jsb.2005.06.001 16043364

[23] A. Patwardhan , J. M. Carazo , B. Carragher , R. Henderson , J. B. Heymann , E. Hill , G. J. Jensen , I. Lagerstedt , C. L. Lawson , S. J. Ludtke , D. Mastronarde , W. J. Moore , A. Roseman , P. Rosenthal , C. O. Sorzano , E. Sanz-Garcia , S. H. Scheres , S. Subramaniam , J. Westbrook , M. Winn , J. R. Swedlow , and G. J. Kleywegt , “ Data management challenges in three-dimensional EM,” Nat. Struct. Mol. Biol. 19(12), 1203–1207 (2012). 10.1038/nsmb.2426 23211764 PMC4048199

[24] A. Patwardhan , A. Ashton , R. Brandt , S. Butcher , R. Carzaniga , W. Chiu , L. Collinson , P. Doux , E. Duke , M. H. Ellisman , E. Franken , K. Grunewald , J. K. Heriche , A. Koster , W. Kuhlbrandt , I. Lagerstedt , C. Larabell , C. L. Lawson , H. R. Saibil , E. Sanz-Garcia , S. Subramaniam , P. Verkade , J. R. Swedlow , and G. J. Kleywegt , “ A 3D cellular context for the macromolecular world,” Nat. Struct. Mol. Biol. 21(10), 841–845 (2014). 10.1038/nsmb.2897 25289590 PMC4346196

[25] R. Henderson , A. Sali , M. L. Baker , B. Carragher , B. Devkota , K. H. Downing , E. H. Egelman , Z. Feng , J. Frank , N. Grigorieff , W. Jiang , S. J. Ludtke , O. Medalia , P. A. Penczek , P. B. Rosenthal , M. G. Rossmann , M. F. Schmid , G. F. Schroder , A. C. Steven , D. L. Stokes , J. D. Westbrook , W. Wriggers , H. Yang , J. Young , H. M. Berman , W. Chiu , G. J. Kleywegt , and C. L. Lawson , “ Outcome of the first electron microscopy validation task force meeting,” Structure 20(2), 205–214 (2012). 10.1016/j.str.2011.12.014 22325770 PMC3328769

[26] R. J. Read , P. D. Adams , W. B. Arendall , A. T. Brunger , P. Emsley , R. P. Joosten , G. J. Kleywegt , E. B. Krissinel , T. Lutteke , Z. Otwinowski , A. Perrakis , J. S. Richardson , W. H. Sheffler , J. L. Smith , I. J. Tickle , G. Vriend , and P. H. Zwart , “ A new generation of crystallographic validation tools for the Protein Data Bank,” Structure 19(10), 1395–1412 (2011). 10.1016/j.str.2011.08.006 22000512 PMC3195755

[27] S. Wasilewski and P. B. Rosenthal , “ Web server for tilt-pair validation of single particle maps from electron cryomicroscopy,” J. Struct. Biol. 186(1), 122–131 (2014). 10.1016/j.jsb.2014.02.012 24582855

[28] I. Lagerstedt , W. J. Moore , A. Patwardhan , E. Sanz-Garcia , C. Best , J. R. Swedlow , and G. J. Kleywegt , “ Web-based visualisation and analysis of 3D electron-microscopy data from EMDB and PDB,” J. Struct. Biol. 184(2), 173–181 (2013). 10.1016/j.jsb.2013.09.021 24113529 PMC3898923

[29] S. Abbott , A. Iudin , P. K. Korir , S. Somasundharam , and A. Patwardhan , “ EMDB web resources,” Curr. Protoc. Bioinf. 61(1), 5.10.11–15.10.12 (2018). 10.1002/cpbi.48 PMC6040833 30008982

[30] C. L. Lawson and W. Chiu , “ Comparing cryo-EM structures,” J. Struct. Biol. 204(3), 523–526 (2018). 10.1016/j.jsb.2018.10.004 30321594 PMC6464812

[31] J. B. Heymann , R. Marabini , M. Kazemi , C. O. S. Sorzano , M. Holmdahl , J. H. Mendez , S. M. Stagg , S. Jonic , E. Palovcak , J. P. Armache , J. Zhao , Y. Cheng , G. Pintilie , W. Chiu , A. Patwardhan , and J. M. Carazo , “ The first single particle analysis map challenge: A summary of the assessments,” J. Struct. Biol. 204(2), 291–300 (2018). 10.1016/j.jsb.2018.08.010 30114512 PMC6205511

[32] B. A. Barad , N. Echols , R. Y. Wang , Y. Cheng , F. DiMaio , P. D. Adams , and J. S. Fraser , “ EMRinger: Side chain-directed model and map validation for 3D cryo-electron microscopy,” Nat. Methods 12(10), 943–946 (2015). 10.1038/nmeth.3541 26280328 PMC4589481

[33] G. Pintilie and W. Chiu , “ Assessment of structural features in Cryo-EM density maps using SSE and side chain Z-scores,” J. Struct. Biol. 204(3), 564–571 (2018). 10.1016/j.jsb.2018.08.015 30144506 PMC6525962

[34] J. H. Mendez and S. M. Stagg , “ Assessing the quality of single particle reconstructions by atomic model building,” J. Struct. Biol. 204(2), 276–282 (2018). 10.1016/j.jsb.2018.09.004 30213768 PMC6201253

[35] M. A. Herzik, Jr. , J. S. Fraser , and G. C. Lander , “ A multi-model approach to assessing local and global cryo-EM map quality,” Structure 27(2), 344–358.E3 (2019). 10.1016/j.str.2018.10.003 30449687 PMC6365196

[36] G. Pintilie , K. Zhang , Z. Su , S. Li , M. F. Schmid , and W. Chiu , “ Measurement of atom resolvability in cryo-EM maps with Q-scores,” Nat. Methods (in press) (2020). 10.1038/s41592-020-0731-1 PMC7446556 32042190

[37] I. S. Gabashvili , R. K. Agrawal , C. M. Spahn , R. A. Grassucci , D. I. Svergun , J. Frank , and P. Penczek , “ Solution structure of the E. coli 70S ribosome at 11.5 A resolution,” Cell 100(5), 537–549 (2000). 10.1016/S0092-8674(00)80690-X 10721991

[38] K. Zhang , H. Zhang , S. Li , G. D. Pintilie , T. C. Mou , Y. Gao , Q. Zhang , H. van den Bedem , M. F. Schmid , S. W. N. Au , and W. Chiu , “ Cryo-EM structures of Helicobacter pylori vacuolating cytotoxin A oligomeric assemblies at near-atomic resolution,” Proc. Natl. Acad. Sci. U. S. A. 116(14), 6800–6805 (2019). 10.1073/pnas.1821959116 30894496 PMC6452728

[39] M. Tagari , R. Newman , M. Chagoyen , J. M. Carazo , and K. Henrick , “ New electron microscopy database and deposition system,” Trends Biochem. Sci. 27(11), 589 (2002). 10.1016/S0968-0004(02)02176-X 12417136

[40] J. B. Heymann and D. M. Belnap , “ Bsoft: Image processing and molecular modeling for electron microscopy,” J. Struct. Biol. 157(1), 3–18 (2007). 10.1016/j.jsb.2006.06.006 17011211

[41] G. Tang , L. Peng , P. R. Baldwin , D. S. Mann , W. Jiang , I. Rees , and S. J. Ludtke , “ EMAN2: An extensible image processing suite for electron microscopy,” J. Struct. Biol. 157(1), 38–46 (2007). 10.1016/j.jsb.2006.05.009 16859925

[42] S. J. Ludtke , C. L. Lawson , G. J. Kleywegt , H. Berman , and W. Chiu , “ The 2010 cryo-EM modeling challenge,” Biopolymers 97(9), 651–654 (2012). 10.1002/bip.22081 22696402

[43] R. Marabini , B. Carragher , S. Chen , J. Chen , A. Cheng , K. H. Downing , J. Frank , R. A. Grassucci , J. Bernard Heymann , W. Jiang , S. Jonic , H. Y. Liao , S. J. Ludtke , S. Patwari , A. L. Piotrowski , A. Quintana , C. O. Sorzano , H. Stahlberg , J. Vargas , N. R. Voss , W. Chiu , and J. M. Carazo , “ CTF challenge: Result summary,” J. Struct. Biol. 190(3), 348–359 (2015). 10.1016/j.jsb.2015.04.003 25913484 PMC4672951

[44] R. Marabini , S. J. Ludtke , S. C. Murray , W. Chiu , J. M. de la Rosa-Trevin , A. Patwardhan , J. B. Heymann , and J. M. Carazo , “ The electron microscopy exchange (EMX) initiative,” J. Struct. Biol. 194(2), 156–163 (2016). 10.1016/j.jsb.2016.02.008 26873784 PMC5093775

[45] A. Patwardhan , R. Brandt , S. J. Butcher , L. Collinson , D. Gault , K. Grunewald , C. Hecksel , J. T. Huiskonen , A. Iudin , M. L. Jones , P. K. Korir , A. J. Koster , I. Lagerstedt , C. L. Lawson , D. Mastronarde , M. McCormick , H. Parkinson , P. B. Rosenthal , S. Saalfeld , H. R. Saibil , S. Sarntivijai , I. Solanes Valero , S. Subramaniam , J. R. Swedlow , I. Tudose , M. Winn , and G. J. Kleywegt , “ Building bridges between cellular and molecular structural biology,” Elife 6, e25835 (2017). 10.7554/eLife.25835 28682240 PMC5524535

[46] A. Kryshtafovych , P. D. Adams , C. L. Lawson , and W. Chiu , “ Evaluation system and web infrastructure for the second cryo-EM model challenge,” J. Struct. Biol. 204(1), 96–108 (2018). 10.1016/j.jsb.2018.07.006 30017700 PMC6205695

[47] “Editorial, Challenges for cryo-EM,” Nat. Methods **15**, 985 (2018). 10.1038/s41592-018-0256-z 30504885

[48] M. Baker , “ Cryo-electron microscopy shapes up,” Nature 561, 565–567 (2018). 10.1038/d41586-018-06791-6 30254359

[49] Y. Zhu , B. Carragher , R. M. Glaeser , D. Fellmann , C. Bajaj , M. Bern , F. Mouche , F. de Haas , R. J. Hall , D. J. Kriegman , S. J. Ludtke , S. P. Mallick , P. A. Penczek , A. M. Roseman , F. J. Sigworth , N. Volkmann , and C. S. Potter , “ Automatic particle selection: Results of a comparative study,” J. Struct. Biol. 145(1–2), 3–14 (2004). 10.1016/j.jsb.2003.09.033 15065668

